# Evaluation of artwork produced by Alzheimer’s disease outpatients in a pilot art therapy program

**DOI:** 10.1590/s1980-57642008dn10200016

**Published:** 2007

**Authors:** Silvia Andreis Witkoski, Márcia Lorena Fagundes Chaves

**Affiliations:** 1 Medical Sciences Post-Graduation Course, Universidade Federal do Rio Grande do Sul School of Medicine, Porto Alegre, Brazil.; 2 Alzheimer’s Disease Center and Neurogeriatric Clinic from the Neurology Service of Hospital de Clinicas de Porto Alegre.

**Keywords:** art therapy, dementia, Alzheimer’s disease

## Abstract

The use of art as therapy for patients with Alzheimer’s disease is characterized by the opportunity of art expression and is conducted with therapeutic purposes. Art in the context of dementia provides a unique window into the cognitive processes of various brain regions.

**Objective:**

To evaluate association between the severity of cognitive deficit and artwork (type, material, and quality) produced by AD patients in a pilot program.

**Methods:**

Eleven patients were evaluated in a weekly quasi-experiment study following 125 sessions of art therapy over a period of 31 months. Patients were diagnosed with Alzheimer’s disease (N=11) according to standard criteria. The Mini Mental State Examination and a battery of cognitive tests were used to assess cognitive deficit.

**Results:**

Different types of artwork were observed during the sessions for most patients. The selection of drawing or modeling showed significant association with severity of cognitive deficit. Type of material, as well as quality of artwork, also showed a similar association with deficit severity.

**Conclusion:**

The significant association between type of work, drawing or modeling, with severity of cognitive impairment could be influenced by a range of damaged cognitive functions (including visuospatial), and by inadequate perception of graphic elements.

## Abstract (pt)

O uso da arteterapia em pacientes com doença de Alzheimer é uma oportunidade de expressão, conduzida com objetivo terapêutico. A arte no contexto da demência fornece uma abertura única para o acesso ao processamento cognitivo com suporte em várias áreas cerebrais.

**Objetivo:**

Avaliar associação entre gravidade do déficit cognitivo e produto artístico (tipo, material, e qualidade) desenvolvido por pacientes com DA num programa piloto.

**Métodos:**

Onze pacientes foram avaliados semanalmente num estudo quase experimento e seguidos por 125 sessões de arte terapia durante um período de 31 meses. Os pacientes receberam diagnóstico de doença de Alzheimer (N=11) de acordo com os critérios padronizados internacionais. O Mini Exame do Estado Mental e uma bateria de testes cognitivos foram usados para avaliar o déficit cognitivo.

**Resultados:**

Tipos diferentes de produtos artísticos foram observados durante as sessões para a maioria dos pacientes. A seleção de desenho ou modelagem mostrou associação significativa com gravidade do déficit cognitivo. Tipo de material bem como qualidade do produto artístico também apresentaram associação similar com gravidade do déficit cognitivo.

**Conclusão:**

A associação significativa entre tipo de produto artístico, desenho ou modelagem, com gravidade do comprometimento cognitivo pode ser influenciada pela variedade de funções cognitivas comprometidas (incluindo a função viso-espacial) e acabar levando à percepção inadequada dos elementos gráficos.

As a uniquely human talent that has evolved from prehistoric cave paintings, art draws on many brain areas responsible for various cognitive processes. The pattern of degeneration in dementia may lead to predictable changes in art. Alzheimer’s disease is a progressive and fatal neurodegenerative disorder manifested by cognitive and memory deterioration, progressive impairment of activities of daily living, and a range of neuropsychiatric symptoms and behavioral disturbances.^1^ The visuospatial deficits in Alzheimer’s disease may also lead to less precision, and attention to spatial relationships. In general, the art of individuals with AD lacks visual precision but can show appealing use of color and form.^2^ In patients with AD and in the case of the artist Willem de Kooning,^3,4^ some of the most artistically successful pieces were produced in the setting of AD. The Alzheimer’s Disease Association has generated highly successful programs for patients with AD, many of whom have never previously painted.^2^ In some cases of frontotemporal dementia, artistic creativity appears anew as the disease develops.^2^ The artwork is approached in a compulsive manner and is often realistic or surrealistic in style. Art in the context of dementia can provide a unique window into the cognitive processes of various brain regions and an opportunity for rehabilitation.

Art therapy is an established mental health profession that uses the creative process of art making to improve and enhance the physical, mental and emotional well-being of individuals of all ages. It is based on the belief that the creative process involved in artistic self-expression helps people to resolve conflicts and problems, develop interpersonal skills, manage behavior, reduce stress, increase self-esteem and self-awareness, and achieve insight.^5^ It can be used with children, adolescents, adults, older adults, groups, and families to assess and treat anxiety, depression, and other mental and emotional problems and disorders; mental illness; substance abuse and other addictions; family and relationship issues; abuse and domestic violence; social and emotional difficulties related to disability and illness; trauma and loss; physical, cognitive, and neurological problems; and psychosocial difficulties related to medical illness. Art therapy programs are found in a number of settings including hospitals, clinics, public and community agencies, wellness centers, educational institutions, businesses, and private practices.^5^

Recreational and occupational therapy are other techniques that have been used for these patients. Occupational therapy improves patients’ daily functioning and reduces the burden on the caregiver, despite the patients’ limited learning ability.^6^ Recreational and occupational activities associated to psychological support in mild to moderate Alzheimer disease have been shown to be effective in reducing caregiver reaction to behavioral symptoms and disruptive behavior. These results partially confirm findings of previous studies, showing that AD patients treated with similar techniques demonstrated an improvement in behavioral disturbances.^7^

The objective of this study was to evaluate the association of artwork and severity of cognitive deficit over 125 art therapy sessions (31 months) in Alzheimer’s disease patients. The primary function was to run a creative art laboratory and to work one on one to facilitate the use of art as a therapeutic intervention with patients. This pilot program was conducted at the Alzheimer’s Disease Center and Neurogeriatric Clinic from Hospital de Clinicas de Porto Alegre, and explored the links between physicians, artists, patients, and families in the creative process.

## Methods

Eleven Alzheimer’s disease patients selected from the Alzheimer’s Disease Center and Neurogeriatric Clinic from Hospital de Clinicas de Porto Alegre (Brazil) were followed weekly in the Art Therapy Special Program for a 31-month period (125 sessions). Alzheimer’s disease was diagnosed according to standard criteria: the DSM-IV criteria for dementia,^8^ and the NINCDS-ADRDA^9^ for probable AD.

Cognitive deficit was evaluated with the Mini Mental State Examination (MMSE)^10,11^ and a battery of cognitive tests: digit span, span, visual recognition span, Wechsler Logical Memory I and II, clock drawing, house drawing, abstraction tests, calculation, famous faces, and praxis.^10-12^ Table 1 displays the demographic data of the sample. An index for severity of cognitive deficit was used (≥3 abnormal tests, according to established cutoff). Education is presented as categories for illiteracy and elementary school (incomplete), elementary and middle school (complete), and high school and college. Under the illiteracy and incomplete elementary school category, only 1 patient was illiterate from the 3 in this schooling group. Patients were selected for this pilot program if they were not familiar with art techniques, where this information was checked with their families.

**Table 1:** Demographic and cognitive data of the sample.

Variables	Frequency
Sex	
Male	5 (45.5%)
Female	6 (54.5%)
Education	
Illiterate and elementary school (incomplete)	3 (27.3%)
Elementary and middle school (complete)	5 (45.5%)
High school and college	3 (27.3%)
Age (mean±SD)	76.27±5.67 (66-84)
Mini Mental State Examination MMSE (mean±SD)	14.55±8.44 (0-26)
Digit span (mean±SD)6 (54.5%)	3.82±3.49 (1-12)
Word span (mean±SD)	2.36±2.34 (0-7)
Famous faces (mean±SD)	9.00±7.07 (0-20)
Clock drawing	1.65±.55 (0-3.00)
House drawing	1.35±.50 (0-3.00)
Visual recognition span (mean±SD)	5.46±3.64 (0-11)
Logic memory I (mean±SD)	1.36±2.42 (0-7)
Logic memory II (mean±SD)	0.36±0.92 (0-3)
Executive function	10 (91%)
Abstraction - Abnormal Calculation	9 (82%)
Apraxia	8 (73%)

In this weekly art program, drawing and modeling were used to allow patients to express themselves by creating images on paper or in modeling clay (argil or plastic clay). The individuals experienced pleasurable, sustained activity while engaged in producing art. Patients were assisted by an art therapist trained for dementia in weekly group sessions (50 to 60 minutes in duration).

The study was approved by the Ethics Committee for Medical Research of Hospital de Clínicas de Porto Alegre. All participants and/or their proxies signed an informed consent before being enrolled onto the study.

Criteria for choice of material were based on availability, common use among those who had never explored artistic abilities, lower risk of intoxication, ease of handling and cleaning from skin and clothes. All artwork was evaluated by a certified art therapist. Drawings were analyzed for their structure in relation to complexity, balance, and precision. They were classified as

1) unidentifiable;
2) totally unstructured and asymmetric;
3) unmistakable, but elements could be missing and symmetry was partial;
4) detailed, complete and symmetric.

Modelings were analyzed according to the three-dimensionality of the form. They were classified as

1) at random (the object resulted in something unidentifiable);
2) horizontal (the object was built horizontally, bi-dimensionally);
3) vertical (the object was clearly vertical but without volume);
4) completely three-dimensional (vertical, with volume and all angles present).

From the movement point of view, modeling and drawings were also classified into static (from the perspective of stillness) and dynamic (when representing action).

### Statistical analysis

Descriptive statistics are presented as means ±SD for parametric variables and frequency (%) for categorical variables. Categorical variables were analyzed with association tests (chi-square with Yates or Fisher correction as appropriate).

## Results

At each session and throughout the 125 sessions, some kind of artwork was performed, albeit drawing, sculpture or both. The type of artwork significantly associated with severity of cognitive impairment in AD patients (chi-square=38.22; p=0.0001) (Table 2). Less impaired patients tended to perform more modeling while the moderate to severe impaired cases carried out more drawings during sessions. Consequently, the choice of material was also associated with severity (p=0.017). Those who were at the mild phases of AD selected modeling material in 70.6% of sessions, while the moderate to severe patients chose drawing objects in 55.7% of sessions.

**Table 2:** Frequency of artwork type produced by patients during the 125 sessions of art therapy according to cognitive deficit (presented as the sum of patients over all sessions).

	Cognitive deficit	
Artwork	Mild	Severe
Drawing	56 (15%)	590 (59%)
Modeling	124 (33%)	14 (1,4%)
Both	195 (52%)	400 (40%)
Total	375 (100%)	1004 (100%)

Chi-square=38.22; p=0.0001

We also observed a significant association between type of modeling clay and sex (Chi-square=10.92; p=0.004), where fifty four percent of the men preferred argil and 61% of women, the plastic clay. Plastic clay was preferred by around 20% of men and argil by around 20% of women, while the same percentage of men and women preferred both materials.

The movement characteristic of the artwork also showed significant association with sex (chi-square=31.96; p=0.0001). Women expressed static forms in 98.5% of their works and men tended to strike a balance between dynamic (44.8%) and static (55.2%). Of the 6 men in the study, only 2 (33%) were mild, and of the 5 women only 1 (20%) was mild. The analysis of this characteristic according to cognitive deficit did not show significant association because 84% of the severely and 71% of the mild impaired patients presented static forms (chi-square=2.76; p=0.097).

Patients with moderate to severe cognitive impairment performed drawings with the following characteristics: simple format, low identification, unstructured and asymmetric. On the other hand, artwork of mild patients was predominantly complex, identifiable, structured and symmetric (Table 3). Severity of cognitive deficit presented association with modeling in three-dimensions (chi-square =48.82; p=0.0001). The shapes of the artworks by moderate to severely impaired patients were 10% at random, 47% horizontal, 10% vertical, and only 33% completely three-dimensional, whereas shapes by less impaired patients were 100% completely three-dimensional.

**Table 3:** Distribution of drawing structure (complexity, precision, and balance) (presented as the sum of patients over all sessions).

Structure	Cognitive deficit	
classification	Mild	Severe
1	0	127 (22%)
2	0	248 (43%)
3	15 (14%)	58 (10%)
4	90 (86%)	144 (25%)
Total	105 (100%)	576 (100%)

Chi-square=48.82; p=0.0001. Structure classification: 1, unidentifiable; 2, totally unstructured and asymmetric; 3, unmistakable, but elements could be missing and symmetry was partial; 4, detailed, complete and symmetric.

## Discussion

This study analyzed association between characteristics of the artwork and severity of cognitive deficit over the course of 125 art therapy sessions (31 months) in Al­zheimer’s disease patients. We ran a creative art laboratory and facilitated the use of art as a therapeutic intervention with patients, exploring the links between physicians, artists, patients, and families in the creative process. The significant association between type of work, drawing or modeling, with severity of cognitive impairment may be related to the nature of the activity itself, as well as to factors directly related to the process of dementia.

Concerning the first hypothesis, drawing is a graphic representation of forms or objects on a surface by means of lines, where depth and volume appear only as illustrations. Mental representations have been investigated by researchers in philosophy, cognitive psychology and - more recently - cognitive science.^13^ Creating a work of art is an irreversible process involving increasing levels of complexity and unpredictable events. Modeling or shaping, on the other hand, is a three-dimensional form from a malleable medium such as clay. This sort of complex sensory-motor activity is characterized by kinetic and visual sensations that coexist with the performance of the artwork.^14^ Visual imagery, more generally visual representation, may not prove to be a unitary concept; rather it may better be viewed as referring to distinguishable subsystems specialized for performing particular aspects of cognitive tasks.^15^

Visuospatial impairment, a dysfunction caused by dementia, could influence drawing and modeling by producing inadequate perception of the elements, besides visual inattention.^16^ The elements that are very sensitive to perception include distance or depth, color, shape and shape constancy (i.e., the capacity to identify an object independent of size, shape, site or different angles of sight), and background.^17^ The severity of the cognitive deficit influenced the selection of activity. Patients less severely impaired preferred modeling, while those more cognitively impaired more often selected drawing.

We also evaluated the artwork by gender. Women expressed their artwork mostly statically (motionless). According to some modern philosophers, women’s development is based on submission and their creativity may reflect this construct.^18,19^ Men, on the other hand, characterized their artwork largely by the sense of action (motion), which could be explained by the same philosophical view, as the result of socially stimulated virility. The choice of modeling material may be explained by the same theory. The modeling clay, more delicate and colorful, attracted more females, while the argil, a rustic material, appealed to men.

Although AD is more common than Frontotemporal dementia (FTD), there is evidence that patients who have developed or maintained artistic talents had a diagnosis of FTD.^20^ The artistic output reported was diverse but shared many features, and despite patients’ progressive cognitive and social impairment, they showed increasing interest in the fine detail of faces, objects, shapes, and sounds.

Our dementia patients lost their ability to maintain their usual activities, to carry out simple tasks, and even their ability to entertain themselves. Nevertheless, we believe that creating opportunities for everyone to explore the possibilities of artistic expression without judgment or criticism may lead to greater self-awareness and self-esteem and can release innate creative energy. An artist designed and implemented each creative project and acted as consultant in how to incorporate it. The basic assumption of this pilot project was that every individual is a natural artist.
